# Assessment of the Preventive Effect of L-carnitine on Post-statin Muscle Damage in a Zebrafish Model

**DOI:** 10.3390/cells11081297

**Published:** 2022-04-11

**Authors:** Joanna Niedbalska-Tarnowska, Katarzyna Ochenkowska, Marta Migocka-Patrzałek, Magda Dubińska-Magiera

**Affiliations:** 1 Hirszfeld Institute of Immunology and Experimental Therapy, The Polish Academy of Sciences, Rudolfa Weigla 12, 53-114 Wroclaw, Poland; joannaniedbalska@gmail.com; 2 Department of Animal Developmental Biology, University of Wrocław, Sienkiewicza 21, 50-335 Wroclaw, Poland; katarzyna.ochenkowska@umontreal.ca (K.O.); marta.migocka-patrzalek@uwr.edu.pl (M.M.-P.)

**Keywords:** lovastatin, L-carnitine, *Danio rerio*, zebrafish, skeletal muscle, cardiac muscle, myotoxicity, statins

## Abstract

Statins, such as lovastatin, are lipid-lowering drugs (LLDs) that have been used to treat hypercholesterolaemia, defined as abnormally elevated cholesterol levels in the patient’s blood. Although statins are considered relatively safe and well tolerated, recipients may suffer from adverse effects, including post-statin myopathies. Many studies have shown that supplementation with various compounds may be beneficial for the prevention or treatment of side effects in patients undergoing statin therapy. In our study, we investigated whether L-carnitine administered to zebrafish larvae treated with lovastatin alleviates post-statin muscle damage. We found that exposure of zebrafish larvae to lovastatin caused skeletal muscle disruption observed as a reduction of birefringence, changes in muscle ultrastructure, and an increase in atrogin-1. Lovastatin also affected heart performance and swimming behaviour of larvae. Our data indicated that the muscle-protective effect of L-carnitine is partial. Some observed myotoxic effects, such as disruption of skeletal muscle and increase in atrogin-1 expression, heart contraction could be rescued by the addition of L-carnitine. Others, such as slowed heart rate and reduced locomotion, could not be mitigated by L-carnitine supplementation.

## 1. Introduction

Statins, e.g., lovastatin, simvastatin, and atorvastatin, belong to a family of lipid-lowering drugs (LLDs) that have been used for many years to combat one of the most common pathological conditions–hypercholesterolaemia, manifested as an elevated level of cholesterol in the patients’ blood. Statins, similar to other LLDs, allow the risk of cardiovascular diseases (CVDs) to be reduced. Statins are considered relatively safe and well-tolerated therapeutic drugs. However, their administration occasionally may induce adverse effects manifested in myotoxicity [1,2,3]. Statins’ mechanism of action is based on the inhibition of 3-hydroxy-3-methylglutaryl coenzyme A reductase (HMGCR), which is the key enzyme of cholesterol synthesis in the mevalonate pathway in the liver. The inhibition of the mevalonate pathway via statins influences the intermediates of cholesterol synthesis, such as coenzyme Q10 (CoQ10) (reviewed by Bouitbir et al. [4]). Statins also influence mitochondrial function, among other ways, by promoting mitochondrial permeability and changing mitochondrial enzyme activities (reviewed by Apostolopoulou et al. [5]).

Molecular mechanisms underlying statin-induced myopathy involve down-regulation of PI3k/Akt signalling and up-regulation of FOXO transcription factor expression, which is associated with increased oxidative stress and inflammation in rat skeletal muscle [6]. Upregulation of expression of the mentioned genes is accompanied by an increase in the transcription of genes, such as atrogin-1 (also known as muscle atrophy F-box, MAFbx), involved in proteasomal- and lysosomal-mediated protein degradation and thus in skeletal muscle atrophy [6,7]. Notably, particular statins, depending on the degree of their lipophilicity, can cause different (or with different severity) side effects [8,9].

Supplementation of statin-exposed individuals with various compounds, including CoQ10, L-carnitine, and geranylgeraniol, has been suggested to be potentially beneficial in preventing or alleviating side effects in patients undergoing statin therapy [8,10,11]. For example, based on the observation that some patients with post-statin myopathy symptoms, such as muscle pain, had L-carnitine-associated abnormalities, it was hypothesised that L-carnitine treatments may alleviate these symptoms [10,12]. Moreover, the muscle-related protective activity of L-carnitine against the toxic effects of simvastatin was observed in rat skeletal muscle [13]. In this case, L-carnitine, acting as a free radical scavenger, was shown to prevent simvastatin-induced impairment of mitochondrial functions triggered by an increase in the generation of superoxide radicals [13].

L-carnitine is an ammonium compound of amino acid origin, naturally occurring in animals. It is involved in energy metabolism via participation in the transport of long-chain fatty acids into mitochondria for their β-oxidation. L-carnitine plays a role in the removal of accumulated toxic fatty acyl-coenzyme A (acyl-CoA) metabolites and maintenance of the balance between free and acyl-CoA [14,15]. In addition, reduced intracellular levels of L-carnitine may lead to the accumulation of lipids in tissues such as the heart, skeletal muscle, and liver, resulting in myopathy and hepatic steatosis [16].

Various studies have revealed that L-carnitine plays an important role in controlling energy metabolism and endurance capacity and can act as an antioxidant [17,18,19]. L-carnitine administration during prolonged physical exercise in mice enhances endurance capacity by promoting fat oxidation and mitochondrial biogenesis [18]. This is accompanied by the accumulation of glycogen stored in skeletal muscle [18]. Supplementation with L-carnitine mitigates oxidative stress during recovery from exercise fatigue, which results in a decrease in exercise-induced muscle damage [17] and attenuates exercise-induced oxidative stress marker levels in resistance-trained athletes [19]. It was also proved that in rats, L-carnitine displays cardioprotective effects against aspartame-induced cardiac toxicity, which may be triggered by the excessive generation of reactive oxygen species, reducing cardiac function [20].

What seems particularly interesting, in the context of preventing muscle damage through supplementation, is the fact that L-carnitine administration impedes muscle atrophy induced by prolonged hindlimb suspension in rats [21]. L-carnitine’s preventive effects are achieved via inhibition of the ubiquitin-proteasome pathway. This is manifested in the suppression of atrogin-1 expression and a decrease in E3 ligase mRNA expression [21]. 

As mentioned above, L-carnitine appears to be a remedy worth considering for alleviating muscle damage of different origins, including post-statin muscle symptoms. However, especially in the case of post-statin muscle damage, to verify and thoroughly understand the mechanism of action of both statins, which trigger muscle damage and L-carnitine as a potential therapeutic, further studies, including research using model organisms such as zebrafish, need to be conducted. 

Zebrafish has been proved as an excellent model for human muscular diseases of different origins (reviewed previously [22,23]), including the LLD-induced myopathies [7,24,25,26,27,28,29] reviewed by Dubińska-Magiera et al. [30]. Studies using a zebrafish model make it possible to understand the mechanisms underlying statin-induced muscle damage [7]. For example, exposure of zebrafish embryos to lovastatin enhances the expression of atrogin-1, which is known to be a crucial protein involved in skeletal muscle atrophy and a component of the ubiquitin-proteasome pathway [7]. Besides stimulation of *atrogin-1* expression, statins may lead to muscle fibre damage, developmental arrest, improper axis elongation, somite compression, changes in the muscle cytoskeleton and myofibril organisation, and pericardial oedema [7,25,29,31]. Recently [28], it was also discovered that exposure of zebrafish embryos to simvastatin or cerivastatin stimulates the expression of glucocorticoid-induced leucine lock (GILZ), which has been indicated as a protein involved in skeletal muscle differentiation [32]. This leads to disruption of embryonic muscle development and muscle contraction impairment [28]. 

Zebrafish was also used to evaluate statins’ influence on heart development and functioning. It was proven that in zebrafish embryos, atorvastatin treatment leads to heart defects followed by pericardiac oedema and impaired cardiac performance manifested in a dose-dependent heart rate decrease [33]. Similar effects were observed in the case of simvastatin administration, which also reduces zebrafish embryos’ heartbeat frequency [26].

In our study, we aimed to verify the hypothesis that the supplementation of zebrafish larvae with L-carnitine reduces cardiac and skeletal muscle damage induced by lovastatin exposure. The obtained results suggest that the muscle-protective effect of L-carnitine is partial. This incomplete action is reflected in L-carnitine’s ability to prevent, among other things, disruption of muscle structure, while having no protective effect on heartbeat rate. Our results provide interesting information which can be used for further investigation of the post-statin muscle damage mechanism and assessment of L-carnitine’s preventive potential.

## 2. Materials and Methods

### 2.1. Ethical Statement

All experiments were carried out following ethical permission approved by the Local Ethics Commission in Wrocław (108/2014), Poland.

### 2.2. Animal Maintenance and Handling

Zebrafish (*Danio rerio*), wild type strains (AB-Tu and Tubingen) and a line with labelled motoneurons Tg (*mnx1*:TaqRFP-T) were used. The latest line was generated by transgenesis of TagRFP-T expressed from zebrafish motor neuron and pancreas homeobox 1 (mnx1) upstream elements (TagRFP-T-red fluorescent protein variant containing a mutation S158T) [34]. If there was no information about the zebrafish line used, the experiment was carried out with the wild line. Zebrafish were raised, staged, and maintained following standard procedures [35,36]. The embryos were obtained by natural spawning and raised at 28 °C with a photoperiod of 14 h light/10 h dark. The larvae were anaesthetized using 0.04% tricaine in embryo medium in the experiments that required it.

### 2.3. Experiment Design and Lovastatin Treatment

Lovastatin (LOV), L-carnitine (LC), and dimethylsulfoxide (DMSO) were purchased from Merck (Darmstadt, Germany). Stock solutions of lovastatin were prepared by dissolving it in DMSO, and the stock solution of L-carnitine was prepared by dissolving it in distilled water. The experimental solutions were obtained by diluting the stock solution in the embryo medium. 

The concentration of lovastatin was selected on the basis of experimental evaluation. To determine the concentration that is lethal to 50% of zebrafish embryos (LD50), toxicological tests were performed on 6-well plates. The volume of each well was 10 mL. Twenty 96hpf embryos were placed in each well. The larvae were incubated with the tested substance for 24 h, and the number of survivors was counted. The LC50 was calculated by plotting the log-concentration of lovastatin versus logits of mortality. According to the LC50 plot, the effective concentration at which 50% of larvae do not survive (logit 0) was 31.5 µM (Supplementary Figure S1). The lovastatin minimal dose was chosen according to Hanai et al., 2007 [7], where authors describe its influence on zebrafish morphology within the range of 0.025 and 5 µM. To observe atrophy in muscles, but to avoid the severe phenotype, we utilise the medium concentration of 0.5 µM lovastatin. 

The minimal efficiency doses of L-carnitine were selected based on published data [37], where researchers show the effects with the use of 0.5 mM (i.e., 500 µM) acetyl L-carnitine. We utilise two experimental doses (100 and 200 µM) to check if we will see the effect in all planned tests. 

We performed experiments with seven investigated groups: NT (non-treated larvae), VC (vehicle control; larvae incubated in solvent, DMSO solution in embryo medium), LOVVLOV (larvae incubated in 0.5 μM lovastatin), LC100 (larvae incubated in 100 μM L-carnitine), LC200 (larvae incubated in 200 μM L-carnitine), LOV+LC100 (larvae incubated in mixture of 0.5 μM lovastatin and 100 μM L-carnitine), and LOV+LC200 (larvae incubated in mixture of 0.5 μM lovastatin and 200 μM L-carnitine). Groups NT, VC, LC100, and LC200 were treated as control groups. To obtain zebrafish embryos for experimental procedures, spawning was carried out in several spawning containers. Fertilised eggs from each spawning container were separately collected, rinsed with embryo medium, allocated to 150 mm Petri dishes filled with freshly prepared embryo medium, and incubated at 28.0 °C under the same photoperiod conditions as the zebrafish stock. Quality of developing embryos was assessed with the use of the dissecting microscope, and embryo medium was renewed daily in order to maintain optimal developing conditions. Non-viable embryos were removed. Then, after pooling larvae from all 150 mm Petri dishes, zebrafish larvae (96 h post-fertilisation [hpf]) were randomly allocated to 50 mm Petri dishes filled with 5 mL per 10 individuals of freshly prepared experimental solutions. Animals were incubated at 28.0 °C for 24 h under standard photoperiod conditions. Larvae (120 hpf) were rinsed with embryo medium and collected for further experiments. The experimenter conducting particular experiments was not involved in collecting the larvae. The experimenter conducting particular experiments (morphology and birefringence assessments) were also blinded to the treatment groups.

### 2.4. Birefringence Assay

Muscle birefringence was analysed in 120 hpf zebrafish larvae (40–86 in each group). Animals were anaesthetized with 0.04% tricaine and placed on a depression glass microscope slide. While the polarising filters were crossed, the fish were rotated to find the angle that maximised birefringence. The microscope exposure was adjusted to see the light refracting through the trunk skeletal muscle of the vehicle control (VC) fish. All settings remained unchanged during the examinations of all control and investigated groups. The observations were performed, and images were acquired using the Leica DM5000 light microscope (Leica, Munich, Germany) with a pair of polarised lenses. ImageJ software was used to quantify the birefringence.

### 2.5. Transmission Electron Microscopy

For electron microscopic techniques, the 120 hpf zebrafish larvae were anaesthetized. Larva fixation, embedding, and sectioning were performed as described previously [38].

### 2.6. RNA Isolation, Reverse Transcription, and Real-Time Quantitative PCR (RT-qPCR)

Total RNA from investigated larvae was extracted using the Extracol reagent (EURX, Gdańsk, Poland), according to the protocol provided by the manufacturer. RNA was quantified using the NanoDrop OneC Spectrophotometer (Thermo Scientific, Waltham, MA, USA). The cDNA was synthesised using a HighCapacity cDNA Reverse Transcription Kit (Applied Biosystems, Bedford, MA, USA).

Real-time quantitative PCR (RT-qPCR) was performed using the CFX Connect Real-Time PCR Detection System (Bio-Rad, Hercules, CA, USA) utilising the PowerUp SYBR Green Master Mix (Thermo Scientific, Waltham, MA, USA) with the gene-specific primers indicated below (Table 1).

The software automatically determined the Ct values. Standard curves for each pair of primers were prepared by serial 5-fold dilutions of the template cDNA followed by the determination of reaction efficiencies. The number of atrogin-1 molecules was determined from the curve and then normalised to the *rpl13a* gene. The normalised number of *atrogin-1* (*fbxo32*) molecules in the samples obtained from the 5 µm LOV treatment group was considered to be 1 arbitrary unit (A.U.).

### 2.7. Behaviour Tests

#### 2.7.1. Spontaneous Displacement Assay

The spontaneous displacement was examined according to a modified method from Xi et al. [39]. Larvae (18–20 in each group) were placed on a lightbox in 50 mm Petri dishes filled with embryo water and allowed to acclimatise for a minimum of 1 min. The spontaneous movement of the larvae was then recorded with a digital video camera at the frequency of 60 frames per second for 10 min. The movies were converted to AVI format and analysed in ImageJ by manually determining the trajectories of movement and measuring the distance travelled by fish. Individual coordinates determined in ImageJ allow one to create tracking graphs using MS Excel. Due to the high diversity of the reactions of larvae within the groups, the activity assessment was made by counting the percentage of individuals in 4 ranges of the distance travelled: no movement (up to 0.5), from 0.5 to 1.5, from 1.5 to 3, and a distance of more than 3 cm. Values are expressed as a percentage of the larvae responding in each range.

#### 2.7.2. Touch-Evoked Response Assay

The response of larvae to tactile stimulation was examined using a method modified by Granato et al. [40]. Larvae were placed individually on a lightbox in a 50 mm Petri dish filled with embryo water and allowed to accommodate for a minimum of 1 min. Larvae’ response to tactile stimulus was assessed by a gentle touch to the tail (two stimuli) and head (single stimuli) with a needle, and the motion was recorded with a digital video camera at the frequency of 60 frames per second. The movies were then analysed. The responses to stimulation were divided into 4 groups: no response, response without escaping (defined as a lateral undulation of the tail immediately after being touched), short distance (the larvae travelled a distance of no more than 20 mm), and long distance (the larvae travelled more than 20 mm). Values are expressed as a percentage of the larvae responding.

### 2.8. Heartbeat Analysis

The zebrafish larvae were anaesthetized and placed on a glass microscope slide. Next, 30-second movies were recorded with a focus on the heart area. The heartbeat analysis was conducted using a light microscope (Leica DM5000, Leica, Munich, Germany) under the 10× objective. The movies were analysed using Image J software with the time series analyser V3 plugin [41]. The obtained results of the dynamic change of the pixel intensity were then analysed in MS Excel in order to identify the intensity peaks corresponding to the successive beats of the larval heart. The result was doubled to obtain the number of beats per minute. Kymographs were generated in MS Excel on the basis of raw results obtained using a time series analyser and counted intensity peaks.

### 2.9. Analysis of Zebrafish Larva Heart Contraction on the Basis of Time-Lapse Images

Zebrafish larvae (5 in each group) were anaesthetized and placed on a glass slide. Videos of heart movements (several consecutive heartbeats, or in the absence of movement, a movie of at least 20 s) were recorded under 4× magnification with a focus on the heart area (fluorescence microscope, Carl Zeiss, Germany, AxioCam MRc5 digital camera). Then the movies were converted to AVI format, and the perimeter of the larva ventricle and atrium in systole and diastole were determined using Image J (3 times for each fish) (Supplementary Figure S2). Three measurements were made in successive film frames for each analysed individual. The percentage difference between the diastolic and systolic circumferences was calculated using the formula: atrial/ventricular contraction [%] = (Cd − Cs)/Cd × 100, where Cd-circumferences of the atrium or ventricular diastole, Cs circumferences of the atrium or ventricular systole and compared between the groups.

#### Statistical Analysis

Data concerning real-time quantitative PCR for *atrogin-1* expression level are given as means and standard deviations, and their significance was determined with Student’s *t*-test. 

Data regarding birefringence analysis and evaluation of heartbeat are given as means and standard deviations and were statistically analysed using the ANOVA test followed by the Games–Howell post-hoc test due to the lack of homogeneity of variance. 

Contraction of the heart muscle based on time-lapse images is given as means and standard deviations. Spontaneous displacement and touch-evoked response assay are expressed as a percentage of the larvae responding. All three tests were statistically analysed using the Kruskal–Wallis test followed by the Pairwise Mann–Whitney post-hoc test.

A significance level of *p* < 0.05 was used in all statistical analyses. At least three independent experiments were carried out. The data analysis for this paper was generated using the Real Statistics Resource Pack software (Release 6.8), copyright (2013–2020) Charles Zaiontz.

## 3. Results

### 3.1. Lovastatin Treatment Disrupts Zebrafish Larva Skeletal Muscle While L-carnitine Has a Protective Effect

**Figure 1:**
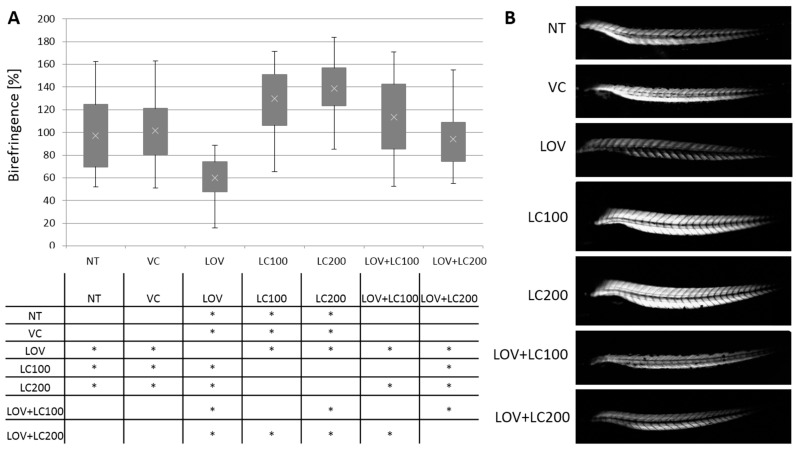
Analysis of skeletal muscle structure of 120 hpf zebrafish larvae exposed to lovastatin (LOV) and L-carnitine (LC) treatment. Control (non-treated, NT; vehicle control, VC; 100 μM L-carnitine, LC100; 200 μM L-carnitine, LC200) were compared with experimental groups (0.5 μM lovastatin, LOV; 0.5 μM lovastatin and 100 μM L-carnitine, LOV+LC100; 0.5 μM lovastatin and 200 μM L-carnitine LOV+LC200). (**A**) The birefringence of zebrafish larvae trunk skeletal muscles, obtained in polarised light, reflects the qualitative changes in muscle structure (magnification 100x, Leica DM5000 light microscope). (**B**) The quantitative analysis revealed that the differences between groups were statistically significant. The table below indicates the pairwise comparison between investigated groups. Statistically significant differences are indicated with *; * *p* < 0.05 (ANOVA test followed by the Games–Howell post-hoc test), the experiment was repeated at least three times (with 40 to 86 individuals in each investigated group). Error bars show the standard deviation.

The 120 hpf zebrafish larva trunk muscle structure was observed and analysed using birefringence, a common non-invasive assay used to determine the degree of muscular disorganisation of zebrafish embryos during early development [42]. The normal muscle structure is visible as bright birefringence, while the disorganisation of the paracrystalline structure of skeletal muscles is manifested in signal reduction. The analysis revealed that the difference between controls and individuals supplemented with lovastatin was statistically significant. The lovastatin-exposed larvae showed a reduction of birefringence, indicating changes in muscle organisation and structure. Moreover, supplementation with both L-carnitine doses (100 and 200 μM) rescued the lovastatin-treated larva phenotype (Figure 1). 

The morphology of most of the larvae treated with both lovastatin and L-carnitine showed no significant changes compared to those of all control groups. Of note, we did not observe any significant changes in analysed zebrafish larva morphology in transmitted light microscopy. Neither changes in the zebrafish trunk nor significant malformations in the pericardial region were observed (Supplementary Figure S3).

### 3.2. Lovastatin Treatment Leads to Sarcomere Malformations in Zebrafish Larva Skeletal Muscle while L-carnitine Has a Protective Effect

**Figure 2:**
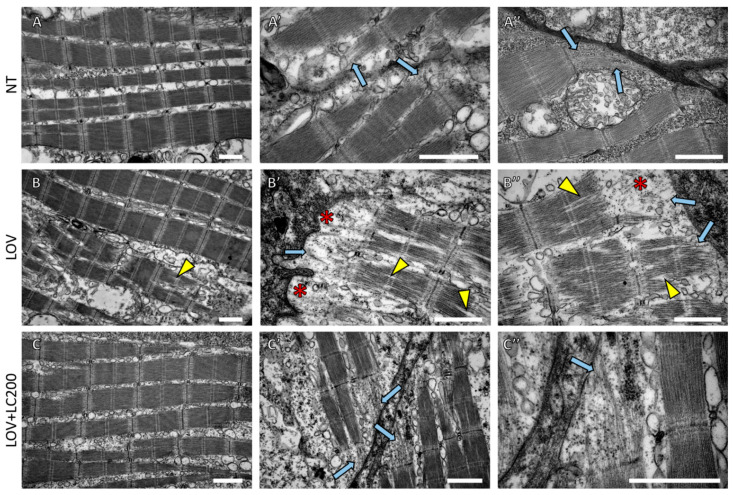
Ultrastructural analysis of skeletal muscles of 120 hpf zebrafish larvae exposed to lovastatin (LOV) and L-carnitine (LOV+LC200) treatment. TEM micrographs show (**A**,**A’**,**A’’**) NT (non-treated larvae); (**B**,**B’**,**B’’**) LOV (0.5 μM lovastatin); (**C**,**C’**,**C’’**) larvae incubated in a mixture of LOV+LC200 (0.5 μM lovastatin and 200 μM L-carnitine). Light blue arrows indicate sarcomeric filaments within subsarcolemmal regions; yellow arrowheads indicate delamination of filaments within sarcomeres; note the disruption of sarcomeric filaments in skeletal muscles of lovastatin-treated larvae (red asterisks); scale bar = 1 µm.

Previous studies on statins’ impact on zebrafish skeletal muscles involved analysis using light microscopy [7,25,26]. To gain further insight into the nature of the observed muscle damage, we decided to undertake a zebrafish lovastatin-treated ultrastructural analysis of skeletal muscles (Figure 2). Our results of TEM phenotype assessment showed that lovastatin exposure triggers contractile apparatus abnormalities. These involve delamination of filaments within sarcomeres and the disruption of sarcomeric filament organisation in the vicinity of the sarcolemma. Interestingly, simultaneous supplementation of zebrafish larvae with L-carnitine at a concentration of 200 μM showed a protective effect manifested in the preservation of a sarcomeric organisation of myofibrils similar to that present in non-treated individuals. This protective effect was also visible in the filaments’ sub-sarcolemmal regions (Figure 2).

### 3.3. Lovastatin Treatment Stimulates Atrogin-1 Expression in Zebrafish Larvae While L-carnitine Has a Protective Effect

**Figure 3:**
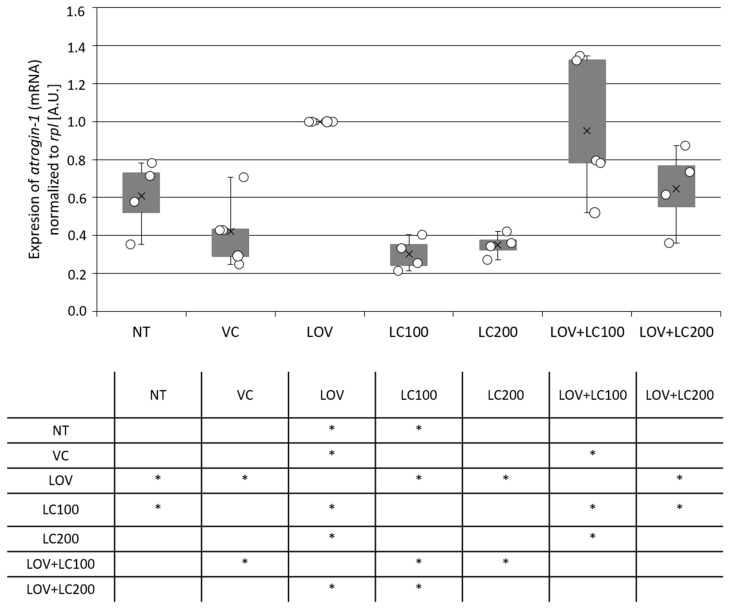
Real-time quantitative PCR (RT qPCR) of *atrogin-1* mRNA expression level in the whole body of 120 hpf zebrafish larvae exposed to lovastatin (LOV) and L-carnitine (LC) treatment. Bar graph demonstrates *atrogin-1* mRNA expression level of zebrafish larvae in control (non-treated, NT; vehicle control, VC; 100 μM L-carnitine, LC100; 200 μM L-carnitine, LC200) and experimental (0.5 μM lovastatin, LOV; 0.5 μM lovastatin and 100 μM L-carnitine, LOV+LC100; 0.5 μM lovastatin and 200 μM L-carnitine LOV+LC200) groups. Expression of atrogin-1 mRNA was normalised to *rpl13a* (ribosomal protein L13a). A.U., arbitrary unit. Error bars show the standard deviation. The tables below indicate the pairwise comparison between *atrogin-1* expression levels in investigated groups. Statistically significant differences are indicated with *; * *p* < 0.05 (Student’s *t*-test). The experiment was performed 3 times (with 25–30 individuals in each investigated group).

Elevated atrogin-1 level is considered an indicator of muscle atrophy. Statin-induced muscle damage has also been associated with an increase in muscle *atrogin-1* expression. This phenomenon was observed in, inter alia, zebrafish embryos exposed to these compounds [7]. Therefore, we decided to investigate whether *atrogin-1* expression is also increased in 120 hpf zebrafish larvae treated with lovastatin. Our results obtained using real-time quantitative PCR (RT-qPCR) confirmed that the mRNA *atrogin-1* expression level increased significantly after lovastatin administration in comparison to all control groups (Figure 3). 

We also evaluated *atrogin-1* mRNA levels in lovastatin-treated groups that received additional L-carnitine supplementation. In this case, we found that only the higher dose of L-carnitine (200 μM) had a significant alleviating effect, which was manifested by inhibiting the increase in the expression of *atrogin-1*, maintaining it at a level similar to those observed in the control groups (Figure 3).

### 3.4. Lovastatin Treatment Alters Swimming Behaviour of Zebrafish Larvae While L-carnitine has no Protective Effect 

**Figure 4:**
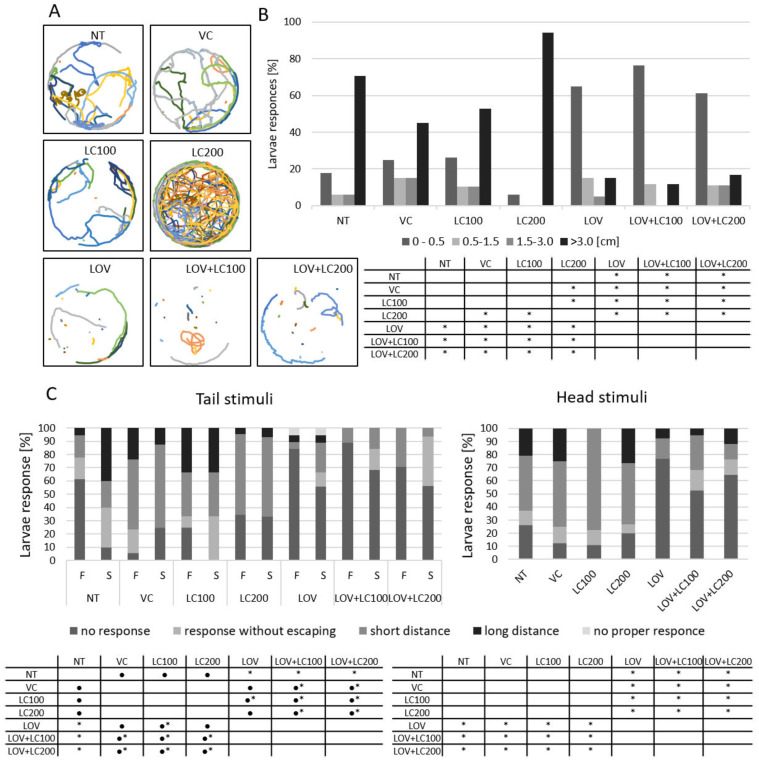
Swimming behaviour of 120 hpf zebrafish larvae exposed to lovastatin (LOV) and L-carnitine (LOV+LC200) treatment. (**A**) Spontaneous displacement of zebrafish larvae in control (non-treated, NT; vehicle control, VC; 100 μM L-carnitine, LC100; 200 μM L-carnitine, LC200) and experimental (0.5 μM lovastatin, LOV; 0.5 μM lovastatin and 100 μM L-carnitine, LOV+LC100; 0.5 μM lovastatin and 200 μM L-carnitine LOV+LC200) groups. Graphs show larvae displacement over the 10 min period. (**B**) Bar graph demonstrates the percentage of zebrafish larvae in seven investigated groups in 4 ranges of the distance travelled: no movement (up to 0.5), from 0.5 to 1.5, from 1.5 to 3, and more than 3 cm. The table below indicates the pairwise comparison between responses of seven investigated groups. Statistically significant differences are indicated with *; * *p* < 0.05 (Kruskal–Wallis test followed by the Pairwise Mann–Whitney post-hoc test). The experiment was repeated at least 3 times (with 18 to 20 individuals in each investigated group). (**C**) Touch-evoked response assay. Stacked column graphs demonstrate the number of zebrafish larvae as a function of their response to first (F) and second (S) tail touch stimulation, as well as in response to a head touch. Depending on the type of reaction, zebrafish larvae were divided into 4 groups: no response, response without escaping (defined as a lateral undulation of the tail immediately after being touched), short distance (larvae travelled a distance of no more than 20 mm), long distance (larvae travelled more than 20 mm). The tables below indicate the pairwise comparison between responses of seven investigated groups. Statistically significant differences are indicated with *; * *p* < 0.05 (Kruskal–Wallis test followed by the pairwise Mann–Whitney post-hoc test). The experiment was repeated at least 3 times (with 18 to 20 individuals in each investigated group).

To further characterise the effects of the test compounds (lovastatin and L-carnitine) on skeletal muscle performance in zebrafish larvae, we carried out swimming behaviour tests (spontaneous larval displacement and response to tactile stimuli) in larvae treated with these substances. Our analyses showed that zebrafish larvae treated with lovastatin exhibited significantly decreased spontaneous larval displacement in comparison to all control groups (Figure 4A,B). Administration of L-carnitine, regardless of the applied dose (100 and 200 μM), showed no therapeutic effect (Figure 4A,B). 

We also examined the response of larvae to tactile stimuli. We observed that the response to tactile stimuli in larvae was significantly decreased, regardless of the method of stimulation (touching the tail or the head), in the lovastatin-treated group in comparison to all controls (Figure 4C). Furthermore, in this test, we observed that L-carnitine treatment, regardless of the dose used, did not improve locomotion in treated individuals (Figure 4C).

### 3.5. Lovastatin Treatment Alters Heartbeat Rate of Zebrafish Larvae While L-carnitine Has no Protective Effect

**Figure 5:**
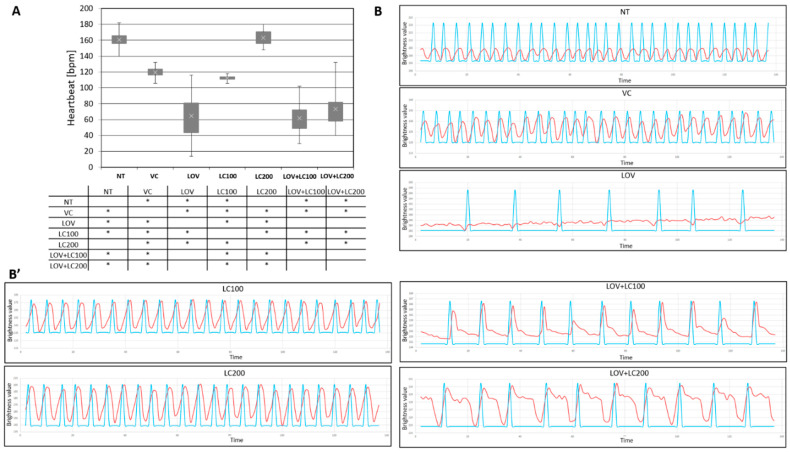
Heartbeat analysis of 120 hpf zebrafish larvae exposed to lovastatin (LOV) and L-carnitine (LC) treatment. (**A**) Bar graph demonstrates heartbeats of zebrafish larvae in control (non-treated, NT; vehicle control, VC; 100 μM L-carnitine, LC100; 200 μM L-carnitine, LC200) and experimental (0.5 μM lovastatin, LOV; 0.5 μM lovastatin and 100 μM L-carnitine, LOV+LC100; 0.5 μM lovastatin and 200 μM L-carnitine LOV+LC200) groups. The table below indicates the pairwise comparison between heartbeat rates of seven investigated groups. Statistically significant differences are indicated with *; * *p* < 0.05 (ANOVA test followed by the Games–Howell post-hoc test). bpm, beats per minute. The experiment was repeated at least 3 times (with 8 to 31 individuals in each investigated group). Error bars show the standard deviation. (**B**,**B’**) exemplary kymographs of investigated groups; red line, dynamic pixel change pattern; blue line, smoothed plot profile. Kymographs were generated via Time Series Analyser 3 (TSA, Image J plugin).

To assess whether lovastatin exposure affects zebrafish larvae (120 hpf) heartbeat performance, we decided to perform analyses that included evaluations of heart rate. Our studies revealed that lovastatin significantly reduces heartbeat rates in comparison to all control groups (Figure 5). 

We also evaluated the heartbeat parameter in relation to L-carnitine supplementation as a substance with the potential to mitigate the negative effects of lovastatin. Our study did not show that L-carnitine had a protective effect on the heartbeat in lovastatin-treated individuals. Administration of L-carnitine at the two investigated concentrations (100 and 200 μM) to lovastatin-administered zebrafish did not help restore the heartbeat rate observed in the control groups. In both tested groups (LOV+LC100 and LOV+LC200), the reduction in heartbeat was significant and similar to that observed in the LOV group (Figure 5).

### 3.6. Lovastatin Treatment Affects Heart Contraction in Zebrafish Larvae While L-carnitine Has a Protective Effect

**Figure 6:**
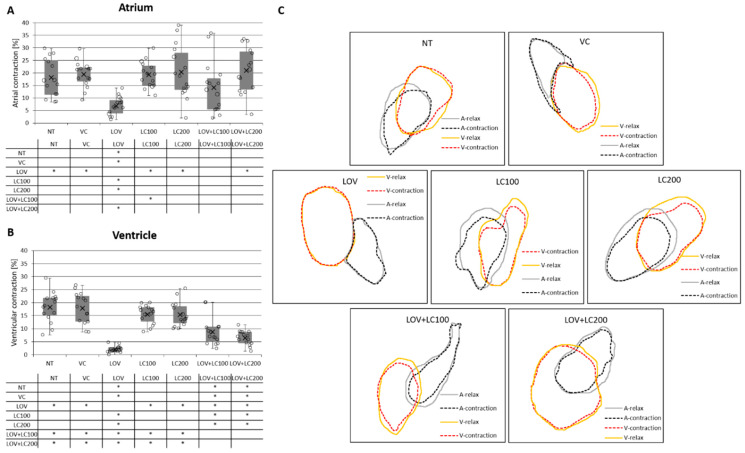
Analysis of heart contraction in 120 hpf zebrafish larvae exposed to lovastatin (LOV) and L-carnitine (LC) treatment. Atrial contraction expressed as the percentage difference between the atrial surface in systolic and relaxed states. Ventricular contraction expressed as the percentage difference between the ventricular surface in systolic and relaxed states. Bar graphs illustrate measurements of the atrial (**A**) and ventricular (**B**) contraction of zebrafish larva heart in control (non-treated, NT; vehicle control, VC; 100 μM L-carnitine, LC100; 200 μM L-carnitine, LC200) and experimental (0.5 μM lovastatin, LOV; 0.5 μM lovastatin and 100 μM L-carnitine, LOV+LC100; 0.5 μM lovastatin and 200 μM L-carnitine LOV+LC200) groups. The tables below indicate the pairwise comparison between atrial (**A**) and ventricular (**B**) contraction of seven investigated groups. Statistically significant differences are indicated with *; * *p* < 0.05 Kruskal–Wallis test followed by pairwise Mann–Whitney post-hoc tests); 5 individuals in each investigated group. Error bars show the standard deviation. (**C**) Exemplary graphs show the systolic and diastolic heart outlines of seven investigated groups used for calculations. Orange line represents ventricle relaxation (ventricular diastole); red dashed line, ventricle contraction (ventricular systole); grey line, atrium relaxation (atrial diastole); black dashed line, atrium contraction (atrial systole).

Further evaluation of the effect of lovastatin exposure on heart performance included analysis of its contraction. Separate analyses of ventricular and atrial contractions were performed. Our studies revealed that lovastatin significantly reduced both atrial and ventricular contractions in comparison to all control groups (NT, VC, LC100, and LC200) (Figure 6). 

We also evaluated ventricular and atrial contractions in relation to L-carnitine supplementation as a substance with the potential to mitigate the negative effects of lovastatin. In this case, our study showed that L-carnitine exposure exhibits a protective effect in lovastatin-treated individuals. Administration of L-carnitine at both investigated concentrations (100 and 200 μM) to lovastatin-exposed larvae positively influenced ventricular contraction, bringing it back to the level observed in the control groups. In contrast, atrial contraction was restored to a level similar to that observed in the control groups in larvae treated with a higher L-carnitine concentration (200 μM; LOV+LC200; Figure 6).

## 4. Discussion

The studies carried out so far by various researchers have clearly shown that zebrafish are a very reasonable choice as a model for research related to post-statin muscle damage [7,11,25,26,27,28,29,33]. Based on available data, we also decided to use this model organism in our experiments, taking into account several important factors when conducting this type of research. For example, it has been confirmed in numerous studies (including those mentioned above) that the type and severity of the effects of statin administration depend on the dose and chemical structure of the particular statin itself (reviewed by Dubińska-Magiera et al. [30]).

Another important factor is the age of the animals. Studies on the effects of various substances, including statins, on the morphology and physiology of the zebrafish organs and systems, are closely dependent on the developmental stage of the animals used in experimental procedures [26,43,44,45]. For example, in the case of the zebrafish heart, it should be considered that although at 48 hpf the major components of the heart are formed (a two-chambered, atrio-ventricular heart), and its localisation resembles the final destination (within the pericardial cavity), the heart is still immature (reviewed by Brown et al. [46]). Therefore, the results obtained using zebrafish at the embryonic (up to 72 hpf) and larval (after 72 hpf) developmental stages may differ significantly. Younger (embryonic stages) individuals are exposed to the risk of developmental disorders resulting inter alia from the inhibition of important signalling pathways, while older (larval stages) individuals, due to the complete or almost complete development of various organs and systems, are not susceptible to the negative impact of the tested substances to the same extent.

In general, the results of our research in the field of assessing the impact of statins, including lovastatin treatment on the structure of zebrafish muscles and heart performance, confirm many of the observations made by other investigators [7,25,26]. However, we found some discrepancies which we would like to discuss. We must emphasise that our intention was to investigate the effects of the tested compounds (lovastatin and L-carnitine) on already developed muscles. Therefore, we used larvae, not zebrafish embryos, for the study. Additionally, the dose of lovastatin we chose significantly influences the structure of the muscles (assessed with the birefringence assay) without causing serious morphological malformations.

Very young embryos (24 hpf) treated overnight with either lovastatin or simvastatin exhibited abnormalities that comprise developmental arrest, improper axis elongation, and compressed somites [31].

Serious morphological malformations were also reported as a result of high doses (0.375 to 1 μM) of simvastatin administration in embryos at the age of 24 or 30 hpf (after treatment at 6 or 11 hpf, respectively) [26]. Furthermore, pericardial oedema was observed in embryos (48 hpf) in response to low dose (0.3 or 0.6 nM) simvastatin treatment [26].

Other research groups have determined that the exposure of zebrafish larvae (80 hpf) only to a relatively high (500 μg/L, ca. 1.2 μM) dose of simvastatin significantly increased the percentage of individuals with abnormal morphology (affecting eyes, tail, and yolk sac structure), as well as pericardial oedema [29]. Administration of lower doses did not induce such dramatic disorders.

In our study, we observed lovastatin-associated skeletal muscle abnormalities, but not to the same extent as researchers who conducted their examination using zebrafish at earlier embryonical developmental stages [7,25,26,31]. The larvae treated with lovastatin (0.5 μM) and/or L-carnitine showed no significant and extensive morphological malformations compared to those of all control groups, including the pericardial region (Supplementary Figure S3). This may be due to the fact that in the development stage, we examined animals less susceptible to disorders related to inhibition of development. The degree of muscular disorganisation was assessed by us using the birefringence assay, which revealed significant differences between controls and individuals administered with lovastatin (Figure 1). This evaluation also confirmed a significant protective effect of L-carnitine. The ameliorating effect of L-carnitine on muscle damage caused by lovastatin may not be specific to lovastatin itself, but rather has a general nature. The positive effect of L-carnitine treatment on muscle structure was indeed observed previously. The supplementation with L-carnitine was shown to be effective in muscle recovery after tissue disruption, occurring due to extensive physical exercise in humans [47,48]. L-carnitine supplementation leads to a reduction of cellular damage markers, such as myoglobin, creatine kinase, and malondialdehyde (MDA) release, as well as to the free radical ratio reduction [47,48,49,50]. Our results are consistent with the previous data indicating the possibility of mitigation of lovastatin-induced muscle damage by other substances, such as those obtained by Cao [27], which showed that mevalonate alleviates lovastatin-induced myofiber damage in zebrafish embryos.

To better understand the nature of lovastatin-induced muscle damage, we decided to perform an examination using the electron microscope. This analysis showed that lovastatin exposure leads to abnormalities of the contractile apparatus (Figure 2). L-carnitine at a higher concentration (200 μM) rescued the lovastatin-induced phenotype. Ultrastructural examination of muscles treated with statins has also been conducted on rabbit [51]. Similar to our analysis, researchers evaluating skeletal muscles of rabbits exposed to statins observed disruption of myofibrils and Z-bands, as well as the presence of autophagic vacuoles and swollen mitochondria. 

We also assessed the muscle damage process at the molecular level by examining via RT-qPCR the expression of *atrogin-1*, considered a useful marker of muscle atrophy. Our RT-qPCR studies revealed that lovastatin enhances the expression of *atrogin-1* in zebrafish larvae (Figure 3). This is in line with data previously acquired by other research teams [7,27]. For example, Hanai [7], in an in vitro study using RTq-PCR, showed that *atrogin-1* mRNA level is increased by lovastatin in a time- and concentration-dependent manner. At the highest used lovastatin dose (10 μM), the increase in *atrogin-1* mRNA level was 6-fold when compared to the control group. The increase in transcript level was mirrored in protein level growth. The authors also observed a two-fold increase in *atrogin-1* expression in the statin-treated patient’s muscle biopsy compared to non-treated individuals. Their investigation of *atrogin-1* expression in zebrafish embryos treated with lovastatin (0.5 μM) for 12 h also revealed its increase.

The data presented in our manuscript suggest that atrogin-1 may be an appropriate marker for lovastatin-mediated muscle atrophy also in the zebrafish larvae. However, since for each sample preparation, we used 30 whole zebrafish larvae, we could only assess the global changes in *atrogin-1* expression without the possibility to distinguish any differences in mRNA expression with consideration to specific tissues, e.g., skeletal or cardiac muscle.

As with other analyses carried out in this work, the second part of our RT-qPCR analysis involved assessing the potentially protective effects of L-carnitine depending on the concentration used. The results show that administration of L-carnitine at a higher dose (200 μM) to lovastatin-exposed larvae inhibits the increase in the expression of atrogin-1 level and maintains it at a level similar to those in the control groups (Figure 3). This is in agreement with studies conducted with rats [21]. Investigations demonstrated that L-carnitine supplementation led to a reduction in *atrogin-1* expression compared to levels that occurred in individuals with symptoms of muscular atrophy induced by prolonged hindlimb suspension [21].

Interesting results were obtained by researchers carrying out studies on zebrafish larvae exposed to atorvastatin and/or CoQ10 [11]. The *atrogin-1* level was differentially affected by atorvastatin exposure, i.e., it was significantly increased at concentrations of 0.081 and 1.8 μM, but significantly decreased at 0.9 μM in comparison to the control group. Atrogin-1 levels showed the same trend following CoQ10 administration, indicating no rescue effect [11]. The authors speculated that differences in *atrogin-1* levels obtained with doses of 0.9 and 1.8 μM could be associated with the dynamic gene expression during larval development [11]. 

To better and more broadly assess the condition of zebrafish skeletal muscles, swimming behaviour tests are often performed. Negative effects of various statins on zebrafish swimming behaviour have been found in both embryos and larvae. For example, a lower dose (0.3 nM) of simvastatin significantly impaired the swimming capacity of 48 hpf zebrafish embryos, while cholesterol was able to compensate for the effects of simvastatin [26]. In turn, larvae treated with atorvastatin exhibited decreased spontaneous larval displacement, and CoQ10 exposure did not restore atorvastatin-induced reduction in spontaneous movement [11]. However, as shown in other analyses conducted by the same authors, atorvastatin administration—resulting in a significant decrease in larval responses to tactile stimuli—could be completely eliminated with CoQ10 treatment [11]. We also conducted swimming behaviour tests. Regarding the effect of lovastatin on zebrafish movements, we obtained results that are in line with the results of other studies [11,26]. Our research confirmed that lovastatin significantly reduces the larvae’s response to tactile stimulation (Figure 4). However, L-carnitine, in this case, showed no therapeutic effect (Figure 4). This may be due to the fact that despite the improvement in muscle conditioning that occurred after L-carnitine administration, as our other tests have shown, different factors underlined the movement disorders, and their elimination cannot be achieved via L-carnitine administration conducted by us.

As mentioned earlier, statins disrupt the function and structure not only of skeletal muscles but also of the heart. One of the potential symptoms of heart failure is pericardial oedema [26,33]. Although we did not find pericardial oedema as the predominant phenotype in our study, we carried out a cardiac performance evaluation of zebrafish larvae treated with the investigated compounds (lovastatin and L-carnitine).

Pericardial oedema was observed in younger embryos (48 hpf) in response to a low dose (0.3 or 0.6 nM) of simvastatin treatment [26]. Furthermore, their heartbeat frequency was reduced in comparison to the control group. The heartbeat simvastatin phenotype could be rescued by excess cholesterol [26]. Moreover, treatment of zebrafish embryos (aged from 18 to 48 hpf) with atorvastatin led to heart defects followed by pericardiac oedema and impaired cardiac performance manifested in a dose-dependent heart rate decrease. A significant reduction was observed at 10 μM atorvastatin. In this case, cholesterol co-administration had a partial rescue effect [33].

The data mentioned above are consistent with our results, which showed that lovastatin significantly lowers heart rate (Figure 5) and also significantly reduces atrial and ventricular contractions compared with the control groups (Figure 6). The part of our study focused on evaluating the protective potential of L-carnitine showed that supplementation only partially rescued the cardiac phenotype, i.e., did not improve heart rate (Figure 5), but maintained heart contraction at a level similar to that observed in the control groups (Figure 6). 

These discrepancies may be due to a too low dose of L-carnitine used in our experiments. Supporting this supposition is the fact that when examining cardiac contraction, we noted that the higher dose (200 μM) had a significant protective effect on both atrial and ventricular contraction, whereas the lower dose (100 μM) was effective only for ventricular contraction (Figure 6).

## 5. Conclusions

Our study showed that the myotoxic effects of lovastatin on 120 hpf zebrafish larvae manifested in a significant reduction of birefringence, changes in muscle ultrastructure, and a significant increase in *atrogin-1* expression in comparison to all control groups. The research established that all observed myotoxic effects could be rescued by the addition of L-carnitine. We also found that lovastatin exposure has a significant impact on larval heart performance tested for the heartbeat frequency and changes in ventricular and atrial contractions. This impact was manifested by a significant decrease in all examined parameters. Intriguingly, our results indicate that, in this case, L-carnitine exhibits only a partially protective effect by rescuing ventricular and atrial contractions without restoring the heartbeat to a normal level. We also discovered that lovastatin exposure changes zebrafish swimming behaviour by reducing the response to tactile stimuli in larvae and that this effect could not be rescued by the addition of L-carnitine.

Although our study contributes to a better understanding of the mechanisms of action of statins showing the myo- and cardiotoxic effects of lovastatin on zebrafish larvae and providing interesting clues to the protective effects of L-carnitine, further studies, focused on, inter alia, mitochondria condition and oxidative stress, as well as on lovastatin-induced cardiotoxicity, are needed to deliver more details on the molecular basis of our observations.

## Figures and Tables

**Table 1:** Sequences of gene-specific primers used in the real-time quantitative PCR analyses.

Target Gene	Accession Number	Seq F (Forward Primer)	Seq R (Reverse Primer)
*rpl13a*	NM_212784.1	CGCTATTGTGGCCAAGCAAG	TCTTGCGGAGGAAAGCCAAA
atrogin-1	NM_200917.1	AAGCTCTGCCAGTATCACTTC	AGTGCAAGGATGGTCTGTATC

## Data Availability

Not applicable.

## Supplementary Materials

The following supporting information can be downloaded at: https://www.mdpi.com/article/10.3390/cells11081297/s1, Figure S1: Zebrafish mortality and LC50 calculation. (A) Percent mortality of zebrafish larvae at 120 hpf after a 24 h exposure (from 96 hpf) to a range of lovastatin (LOV) concentrations. (B) Logit analysis of the mortality data in (A). The extrapolated LC50 (logit value of 0) is indicated by a line corresponding to an LOV concentration of 31.5 μM. In panel A the data are presented as means ± SEM (n = 20 for each LOV concentration); Figure S2: Analysis of zebrafish larva heart contraction on the basis of time-lapse images. Exemplary measurement of 120 hpf zebrafish larva heart contraction on the basis of time-lapse images (A) Representative image of Tg(mnx1:TaqRFP-T) transgenic zebrafish larvae. (B) Exemplary image, of the control (not treated, NT) and test (incubated in 0.5 μM lovastatin, LOV) zebrafish larva heart (fluorescence microscope, Carl Zeiss, Germany, AxioCam MRc5 digital camera). The “find edges” function in Image J software was used for better contrasting of the heart walls. Images show ventricle and atrium in systole and diastole state. The dashed yellow line shows how the ventricular and atrial volumes were measured. The drawings in black frames show the overlapping of lines during systole (red) and diastole (black); Figure S3: Analysis of morphology of 120 hpf zebrafish larvae exposed to lovastatin (LOV) and L-carnitine (LC) treatment. The morphology of control (non-treated, NT; vehicle control, VC; 100 μM L-carnitine, LC100; 200 μM L-carnitine, LC200) and experimental (0.5 μM lovastatin, LOV; 0.5 μM lovastatin and 100 μM L-carnitine, LOV+LC100; 0.5 μM lovastatin and 200 μM L-carnitine LOV+LC200) individuals was compared using light microscopy. The 120 hpf larvae were anesthetized with 0.04% tricaine, placed on a depression glass microscope slide, and photographed (magnification 100x, Olympus SZ61 dissecting microscope). No distinct alterations in larva morphology were observed. (A) Morphology of representative individuals (B) Percentage analysis of phenotype distribution (normal phenotype; abnormalities in tail region—defined as a curved tail; abnormalities in pericardial area—defined as a slight enlargement of pericardial cavity). The experiment was repeated at least 3 times (with 29 to 40 individuals in each investigated group).
