# Supplementary material for: Assessment of the Preventive Effect of L-carnitine on Post-statin Muscle Damage in a Zebrafish Model
Source: Cells. 2022 Apr 11;11(8):1297. doi: 10.3390/cells11081297 (PMC9032104; doi:10.3390/cells11081297)
Supplement: Supplementary file 1 [file cells-11-01297-s001.zip › cells-1617466-supplementary.pdf]

Supplementary Materials

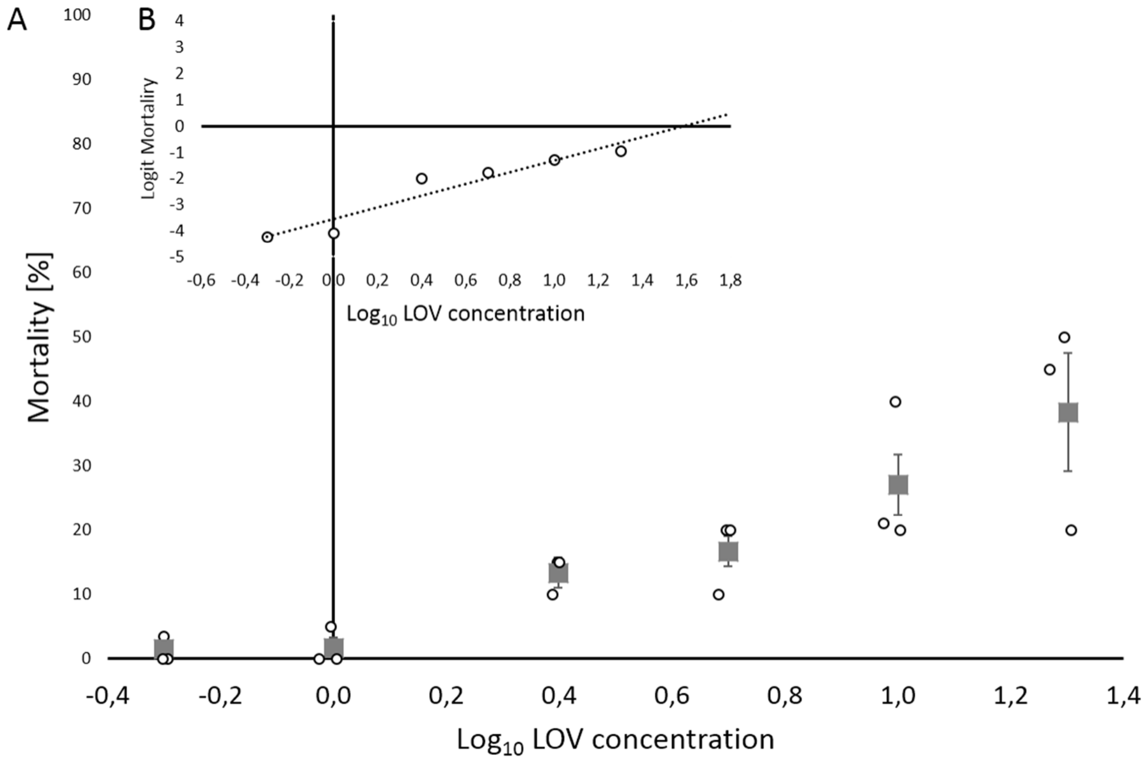

Figure S1. Zebrafish mortality and LC50 calculation.

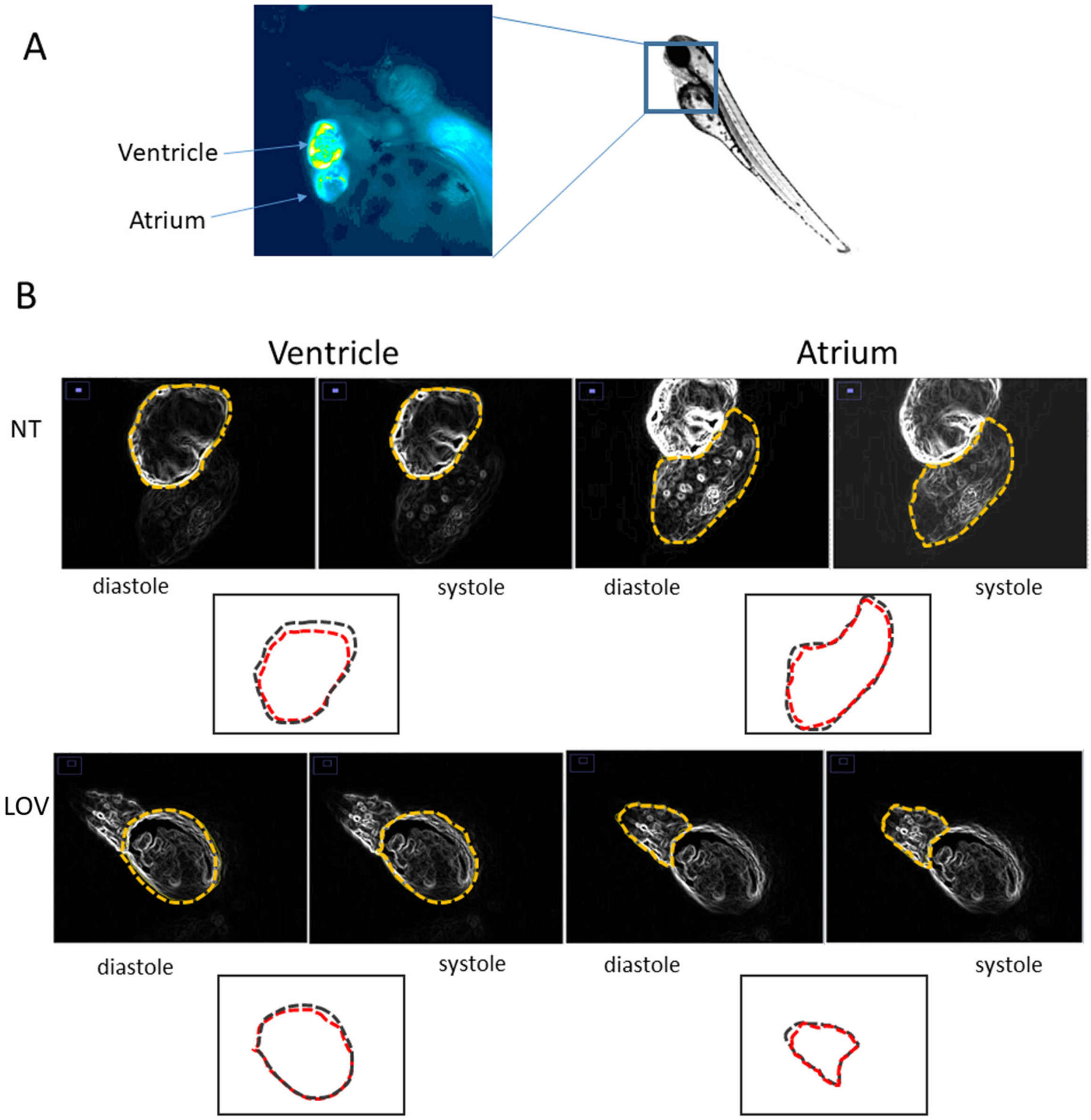

Figure S2. Analysis of zebrafish larva heart contraction on the basis of time-lapse images.

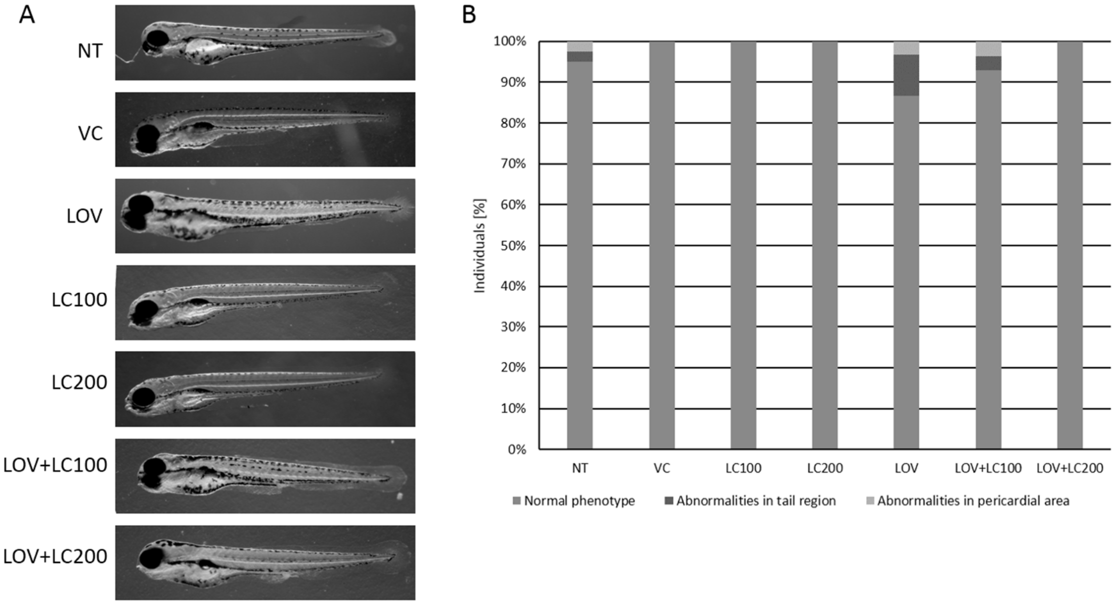

Figure S3. Analysis of morphology of 120 hpf zebrafish larvae exposed to lovastatin (LOV) and L-carnitine (LC) treatment.
